# Short-term spheroid culture of primary colorectal cancer cells as an *in vitro* model for personalizing cancer medicine

**DOI:** 10.1371/journal.pone.0183074

**Published:** 2017-09-06

**Authors:** Maria Jeppesen, Grith Hagel, Anders Glenthoj, Ben Vainer, Per Ibsen, Henrik Harling, Ole Thastrup, Lars N. Jørgensen, Jacob Thastrup

**Affiliations:** 1 Digestive Disease Center, Bispebjerg Hospital, University of Copenhagen, Copenhagen, Denmark; 2 2cureX, Birkerød, Denmark; 3 Department of Pathology, Rigshospitalet, University of Copenhagen, Copenhagen, Denmark; 4 Department of Pathology, Hvidovre Hospital, University of Copenhagen, Copenhagen, Denmark; 5 Department of Drug Design and Pharmacology, University of Copenhagen, Denmark; Chang Gung University, TAIWAN

## Abstract

Chemotherapy treatment of cancer remains a challenge due to the molecular and functional heterogeneity displayed by tumours originating from the same cell type. The pronounced heterogeneity makes it difficult for oncologists to devise an effective therapeutic strategy for the patient. One approach for increasing treatment efficacy is to test the chemosensitivity of cancer cells obtained from the patient’s tumour. 3D culture represents a promising method for modelling patient tumours *in vitro*. The aim of this study was therefore to evaluate how closely short-term spheroid cultures of primary colorectal cancer cells resemble the original tumour. Colorectal cancer cells were isolated from human tumour tissue and cultured as spheroids. Spheroid cultures were established with a high success rate and remained viable for at least 10 days. The spheroids exhibited significant growth over a period of 7 days and no difference in growth rate was observed for spheroids of different sizes. Comparison of spheroids with the original tumour revealed that spheroid culture generally preserved adenocarcinoma histology and expression patterns of cytokeratin 20 and carcinoembryonic antigen. Interestingly, spheroids had a tendency to resemble tumour protein expression more closely after 10 days of culture compared to 3 days. Chemosensitivity screening using spheroids from five patients demonstrated individual response profiles. This indicates that the spheroids maintained patient-to-patient differences in sensitivity towards the drugs and combinations most commonly used for treatment of colorectal cancer. In summary, short-term spheroid culture of primary colorectal adenocarcinoma cells represents a promising *in vitro* model for use in personalized medicine.

## Introduction

Cancer remains a major cause of death in developed countries despite significant progress in understanding the biology of cancer and development of molecular targeted therapies [1–3]. Tumours originating from the same cell type display molecular and functional heterogeneity, which represents an obstacle for developing new drugs and predicting likely responders [3,4]. Currently, clinicians rely on histopathological staging of the tumour [5,6] plus a limited number of molecular tests [7], when devising a therapeutic strategy for each patient. Great effort has been put into identifying biomarkers that can predict clinical response to specific treatment regimens, but few have demonstrated sufficient precision for use in clinical practice [8]. One major reason for the shortcomings of this approach lies within the complexity of the signalling networks that drive tumour growth [9]. Functional assessment of the individual tumour is therefore believed to be of greater clinical value than the molecular footprint.

A promising approach is to test the therapeutic response of cancer cells obtained from the patient’s own tumour towards a variety of drugs. Viable cancer cells can be isolated from freshly obtained tumour tissue and subsequently exposed to therapeutic drugs under controlled experimental conditions [10–15]. Methods for propagating primary cancer cells in the laboratory range from conventional, 2D cell monolayers to more advanced 3D culture systems [16] as well as engraftment of tumour tissue into immunodeficient rodents as patient-derived tumour xenografts [17]. Extensive evidence demonstrates that 3D culture of cancer cells mimics *in vivo* tumour conditions more closely than conventional 2D culture with respect to: 1) cell morphology and organisation [18,19], 2) cell hierarchy and heterogeneity [20,21], 3) protein and gene expression patterns [20,22], 4) growth patterns and distribution of proliferating and apoptotic cells [20,23], 5) cell-cell and cell-matrix interactions [18,19] and 6) metabolic gradients of for example oxygen and drug penetration [24–26]. Patient-derived tumour xenografts also recapitulate many features of the original tumour [17], but this technique needs considerable amounts of tumour tissue, requires several months to evaluate therapeutic response and is very costly [27]. 3D culture systems have potential to assess therapeutic response faster and at a lower cost than PDTX models, while retaining important features and functionalities of the original tumour.

Colorectal cancer is one of the most frequently diagnosed cancers with more than 1.2 million new annual cases worldwide [1]. Despite aggressive multidisciplinary therapy, the overall 5-year survival is only about 60% in the Western World [28,29]. The need for optimization of chemotherapy is therefore pivotal, and we set out to validate a 3D culture system for *in vitro* chemosensitivity testing of primary colorectal cancer cells. Several research groups have previously demonstrated that fragments of human colorectal tumours form rounded multicellular structures in 3D culture, termed “spheroids” or “organoids”, and that these structures can be propagated *in vitro* [30–33]. Chemosensitivity testing requires a robust method with a high success rate for isolation of cancer cells from primary tumour tissue. In order to achieve strong correlation between the test results and the clinical response, the isolated cells should retain essential characteristics of the original tumour.

The aim of the current study was to evaluate how closely primary colorectal cancer cells maintained in short-term 3D culture as spheroids resemble the original tumour and whether the spheroids are suitable for chemosensitivity testing. We established colorectal spheroid cultures from human tumour tissue and characterized the cultures in relation to the original tumours. The spheroids were exposed to standard colorectal chemotherapeutic regimens and response profiles were determined for cultures from different patients.

## Materials and methods

### Patient samples

Tissue samples were collected from 22 patients with colorectal cancer undergoing surgical resection of their primary tumour at Bispebjerg Hospital and Hvidovre Hospital, Copenhagen, Denmark. Furthermore, tissue samples were collected from three patients with metastatic colorectal cancer undergoing surgical resection of their liver metastases at Rigshospitalet, Copenhagen, Denmark. Fresh tumour tissue was collected by a pathologist before routine processing of the specimen. Collected tumour tissue was placed in cold PBS with antibiotics (500 U/ml penicillin, 500 μg/ml streptomycin, 100 μg/ml gentamicin and 2.5 μg/ml amphotericin B) and transported to the laboratory on ice. Tumours other than adenocarcinomas were excluded from the study after receiving the pathology report. The study protocol was approved by the Regional Committee on Health Research Ethics—Capital Region of Denmark (protocol no. H-1-2011-125) and written informed consent was obtained from all patients.

### Spheroid preparation and culture

Spheroids were prepared using a modified version of the protocol published by Kondo et al. [31]. Tumour tissue was washed in PBS with antibiotics, visible fatty and necrotic areas were removed with a scalpel and the tissue was minced into 1–2 mm pieces. Tissue was digested with 1 mg/ml collagenase type II (Gibco, Thermo Fisher Scientific, Waltham, MA, USA) in PBS with antibiotics for 20 min at 37°C. The tissue suspension was filtered sequentially through the following filters: 230 μm mesh filter (Sigma-Aldrich, St. Louis, MO, USA), 100 μm cell strainer (BD Biosciences, Franklin Lakes, NJ, USA), 40 μm cell strainer (BD Biosciences, Franklin Lakes, NJ, USA),) and 30 μm pre-separation filter (MACS, Miltenyi Biotec, Bergisch Gladbach, Germany). Tissue retained by the 230 μm filter was collected and redigested for 10 min at 37°C and passed through the filters again. This step was repeated until all tissue passed through the 230 μm filter. Retained tumour fragments were collected from the 100 μm, 40 μm and 30 μm filters, separating released fragments into three fractions according to size. The isolated tumour fragments were seeded in stem cell medium (StemPro hESC SFM, Thermo Fisher Scientific, Waltham, MA, USA) supplemented with antibiotics (200 U/ml penicillin, 200 μg/ml streptomycin, 100 μg/ml gentamicin and 2.5 µg/ml amphotericin B) in petri dishes coated with agarose (Sigma-Aldrich, St. Louis, MO, USA) and cultured at 37°C in a 5% CO_2_ humidified incubator (MCO-19AIC(UV), Panasonic, Hägersten Sweden).

### Spheroid forming efficacy and culture success

After 3 days of culture, the isolated tumour fragments were inspected under the microscope and their spheroid forming efficacy evaluated. For each size fraction, 50 tumour fragments were examined by light microscopy (Diaphot 200, Nikon, Birkerød, Denmark) at 100x magnification and scored according to whether they had formed spheroids (rounded, smooth surface without clearly defined individual cells) or not (rippled, rough surface with clearly defined individual cells). Spheroid forming efficacy across size fractions was compared by one-way repeated measures ANOVA. Culture success was evaluated by counting the number of formed spheroids. Tumour fragments were aspirated, washed in PBS and filtered to remove single cells and debris. The retained spheroids were counted by light microscopy at 40x magnification. Spheroid preparations with a total number of 150 spheroids or more were considered successful.

### Spheroid growth

After 3 days of culture, spheroids were washed in PBS, filtered and resuspended in fresh stem cell medium. Spheroids were mixed 1:1 with Matrigel (BD Biosciences, Franklin Lakes, NJ, USA) and seeded in 96-well plates coated with agarose at a density of approximately 25 spheroids per well. Plates were incubated for 30 min at 4°C followed by polymerisation of Matrigel for 30 min at 37°C. The spheroids were cultured for 7 days at 37°C in a 5% CO_2_ humidified incubator and light microscopy images were obtained with a digital camera (MU300, AmScope, Irvine, CA, USA) of individual wells every day. Spheroid areas were measured on the obtained images using ImageJ (Rasband, W.S., ImageJ, U. S. National Institutes of Health, Bethesda, Maryland, USA, http://imagej.nih.gov/ij/, 1997–2014). Relative changes in spheroid size during 7 days of culture were assessed by RMANOVA and one-way repeated measures ANOVA.

### Formalin-fixed paraffin-embedded sections of tumour tissue and spheroids

Resected colorectal tissue was fixed in 4% formalin for a minimum of 48 h and cut into tissue blocks. Spheroids were fixed in 4% formalin O/N at 4°C and embedded in 2% agarose. After dehydration in graded alcohol and xylene, tissue blocks and spheroid-containing agarose discs were embedded in paraffin. Sections of 2–4 μm were cut using a microtome (HM 450, Microm, Thermo Fisher Scientific, Waltham, MA, USA) and mounted on FLEX IHC microscope slides (Dako, Glostrup, Denmark). Paraffin sections were heated for 30 min at 60°C (Function Line UT12, Heraeus Instruments, Hanau, Germany) and stored at 4°C until staining.

### Histological staining

Hematoxylin and eosin (H&E) staining and periodic acid Schiff **(**PAS) staining of paraffin sections were performed according to standard protocols. Stainings were visualised by light microscopy (BX51, Olympus, Ballerup, Denmark) and images obtained with digital camera (UC30, Olympus, Ballerup, Denmark).

### Immunostaining

Deparaffinisation of sections and antigen retrieval were performed by pretreatment in PT Link (Dako, Glostrup, Denmark). Sections were pretreated in EnVision FLEX Target Retrieval Solution HigH pH (Dako, Glostrup, Denmark), except for staining with fibroblast antibody, which required pretreatment in EnVision FLEX Target Retrieval Solution Low pH (Dako, Glostrup, Denmark). Sections were blocked and permeabilised in PBS with 2% fetal calf serum, 1% bovine serum albumin (BSA) and 0.25% Triton X-100 for 30 min. Primary antibodies diluted in incubation buffer (PBS with 1% BSA and 0.25% Triton X-100) were applied to sections and incubated for 1 h at RT. The used primary antibodies were monoclonal rabbit-anti-epithelial cell adhesion molecule (EpCAM) (clone E144, Abcam, Cambridge, United Kingdom) diluted 1:400, monoclonal mouse-anti-cytokeratin 20 (clone Ks20.8, Dako, Glostrup, Denmark) diluted 1:100, monoclonal mouse-anti-carcinoembryonic antigen (CEA) (clone II-7, Dako, Glostrup, Denmark) diluted 1:50, monoclonal mouse-anti-fibroblasts (clone TE-7, Millipore, Billerica, MA, USA) diluted 1:100, monoclonal mouse-anti-Ki67 (clone MIB-1, Dako, Glostrup, Denmark) diluted 1:100, monoclonal mouse-anti-ABCB1 (P-glycoprotein) (clone JSB-1, Abcam, Cambridge, United Kingdom) diluted 1:50 and monoclonal mouse-anti-ABCG2 (breast cancer resistance protein) (clone BXP-21, Abcam, Cambridge, United Kingdom) diluted 1:100. Secondary antibodies diluted in incubation buffer were applied to sections and incubated for 30 min at RT. The used secondary antibodies were goat-anti-rabbit Alexa 546 and goat-anti-mouse Alexa 488, both diluted 1:400. Sections were stained with 3 μM Hoechst 33258 (Sigma-Aldrich, St. Louis, MO, USA) in PBS for 5 min and coverslips were mounted with fluorescence mounting medium (Dako, Glostrup, Denmark). Negative control sections were obtained by omission of primary antibody. Stainings were visualised by fluorescence microscopy (Axio Lab.A1, Zeiss, Birkerød, Denmark) and images obtained with digital camera (DMK 72AUC02, The Imaging Source, Bremen, Germany). The number of stained cells was analysed on the obtained images using Image J. The percentage of stained cells was compared by paired-samples t test, Wilcoxon signed-rank test or by one-way repeated-measures ANOVA.

### Terminal deoxynucleotidyl transferase dUTP nick end labelling (TUNEL) staining

Deparaffinisation of sections and antigen retrieval was performed by pretreatment in PT Link in EnVision FLEX Target Retrieval Solution HigH pH. TUNEL staining was performed using the ApopTag Fluorescein In Situ Apoptosis Detection Kit (Millipore, Billerica, MA, USA) according to the manufacturer’s instructions for fluorescent staining of paraffin-embedded tissue. Positive control sections were obtained by pretreatment with 1 μg/ml DNase I (Sigma-Aldrich, St. Louis, MO, USA) in PBS with 4 mM MgCl_2_ for 10 min at RT. Negative control sections were obtained by omission of TdT enzyme. Co-staining with EpCAM was performed as described in the previous section. Stainings were visualised by fluorescence microscopy and images obtained. The number of stained cells was analysed on the obtained images using Image J. The percentage of stained cells was compared by paired-samples t test.

### Chemosensitivity testing

After 3 days of culture, spheroids were washed in PBS, filtered, resuspended in fresh stem cell medium and counted. For each screen, approximately 1000 spheroids were added to an IndiTreat™ screening array (2cureX, Birkerød, Denmark) containing concentration gradients of 5-FU, oxaliplatin, SN38 (the active metabolite of Irinotecan) (Sigma-Aldrich, St. Louis, MO, USA) and combination treatments FOLFOX (5-FU + oxaliplatin) and FOLFIRI (5-FU and SN38). The arrays were scanned on screening day 0, 4 and 7 using an oCelloScope system (Phillips BioCell, Allerød, Denmark). The obtained images were analysed for changes in spheroid area using proprietary Phillips BioCell and 2cureX algorithms. For each well, the relative growth inhibition was calculated by dividing the total spheroid area with the area of the same well at day 0 and the average of the negative controls on the same day as the measurement day. Dose response curves, adjusted r^2^ values and ED25 values were plotted and calculated using Matlab (MathWorks, Natik, MA, USA). Less than *lowest dose* or higher than *highest dose* was used in cases where ED25 values were calculated to be outside the used compound concentration ranges.

### Statistical analysis

All statistical analyses were performed in IBM SPSS statistics 20 (Armonk, New York, USA). A p-value ≤ 0.05 was considered statistically significant.

## Results

### Spheroid cultures can be established from colorectal adenocarcinomas with a high success rate

For 20 tumour samples, the efficiency of culture establishment was evaluated. Two neuroendocrine carcinomas were excluded from the study after receiving the histopathological reports. For tumours diagnosed as adenocarcinomas (n = 18), spheroid cultures were successfully established for 15 (83%) of the obtained samples. One of these tumours was classified as a mucinous adenocarcinoma. The clinical characteristics of the tumours are listed in S1 Table.

### Colorectal spheroids consist of epithelial cells with little fibroblast contamination

**Fig 1:**
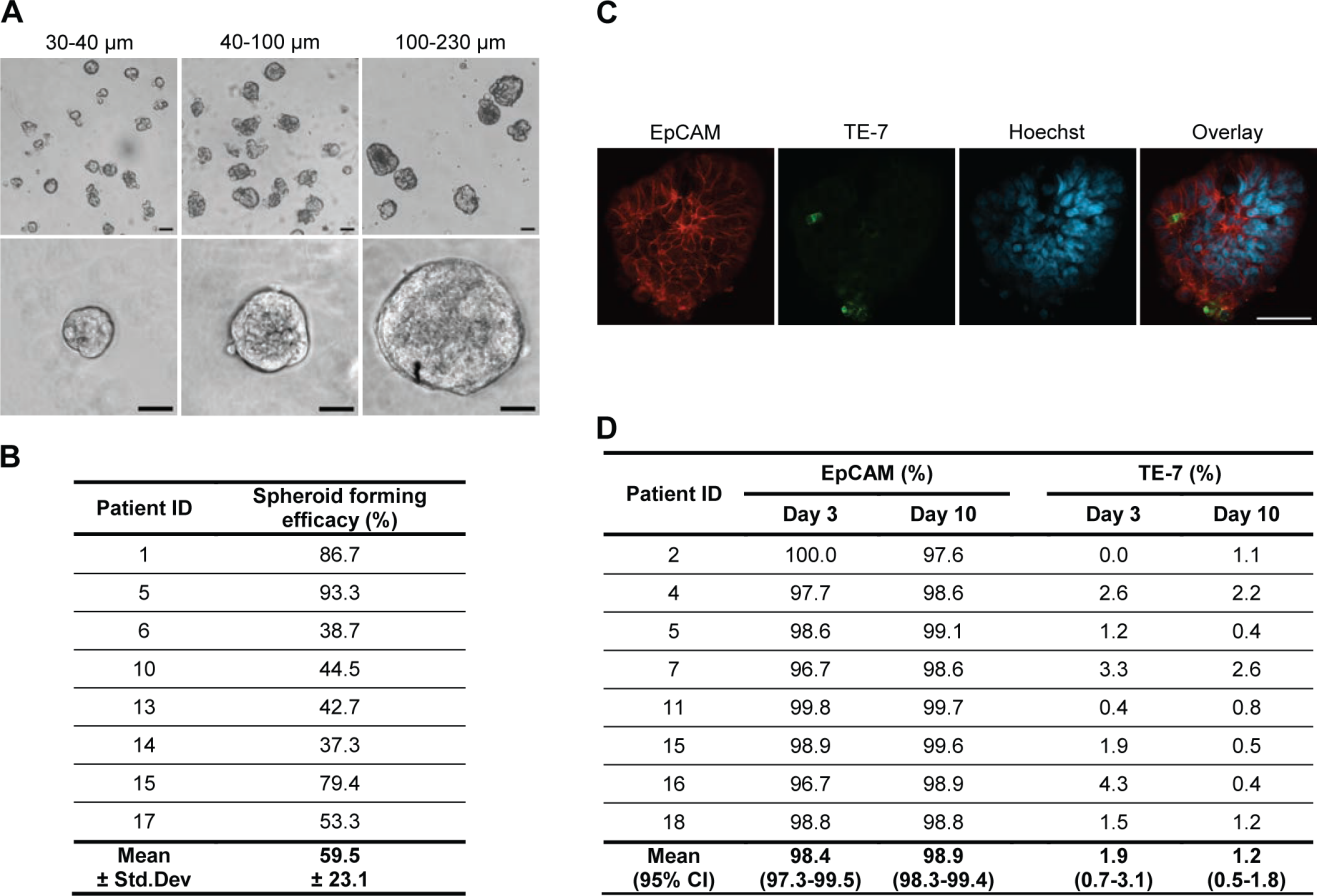
Colorectal spheroid cultures predominantly consist of epithelial cells. (A) Spheroids of three different sizes at low and high magnification after 3 days of culture. Size bar = 50 μm. (B) Spheroid forming efficacy of isolated tumour fragments. Spheroid forming efficacy was defined as the percentage of isolated tumour fragments that had formed spheroids within 3 days of culture. (C) Immunostaining of spheroids for epithelial cell marker EpCAM (red) and fibroblast marker TE-7 (green) after 10 days of culture. Nuclei are stained with Hoechst (blue). Size bars = 50 μm. (D) No significant difference in percentage of spheroid cells stained for EpCAM (p = 0.387) and TE-7 (p = 0.196) at day 3 and day 10 was observed.

Three different sizes of spheroids were prepared for each tumour in order to investigate if the size of the isolated tumour fragments affects spheroid formation and cellular characteristics of spheroids (Fig 1A). Despite individual variation in spheroid forming efficacy between tumours, varying from 40% to more than 90% (Fig 1B), no significant difference in spheroid forming efficacy was found for the three fragment sizes (mean with 95% CI: 100–230 μm = 56.8% (36.6–76.9), 40–100 μm = 62.5% (42.6–82.4), 30–40 μm = 60.5% (40.3–80.6); p = 0.054).

The cell composition of spheroids was assessed by immunostaining for the epithelial marker EpCAM and the fibroblast marker TE-7 after establishment of cultures (day 3) and after short-term culture (day 10). Staining revealed that the spheroids consisted of an almost pure population of epithelial cells with very little fibroblast contamination (Fig 1C). The percentage of spheroid cells stained for EpCAM ranged from 96.7% to 100%, whereas the percentage stained for TE-7 ranged from 0% to 4.3% (Fig 1D). The proportion of EpCAM- and TE-7-positive cells did not change significantly from day 3 to day 10 in culture.

### Colorectal spheroids grow and maintain viability in short-term culture

**Fig 2:**
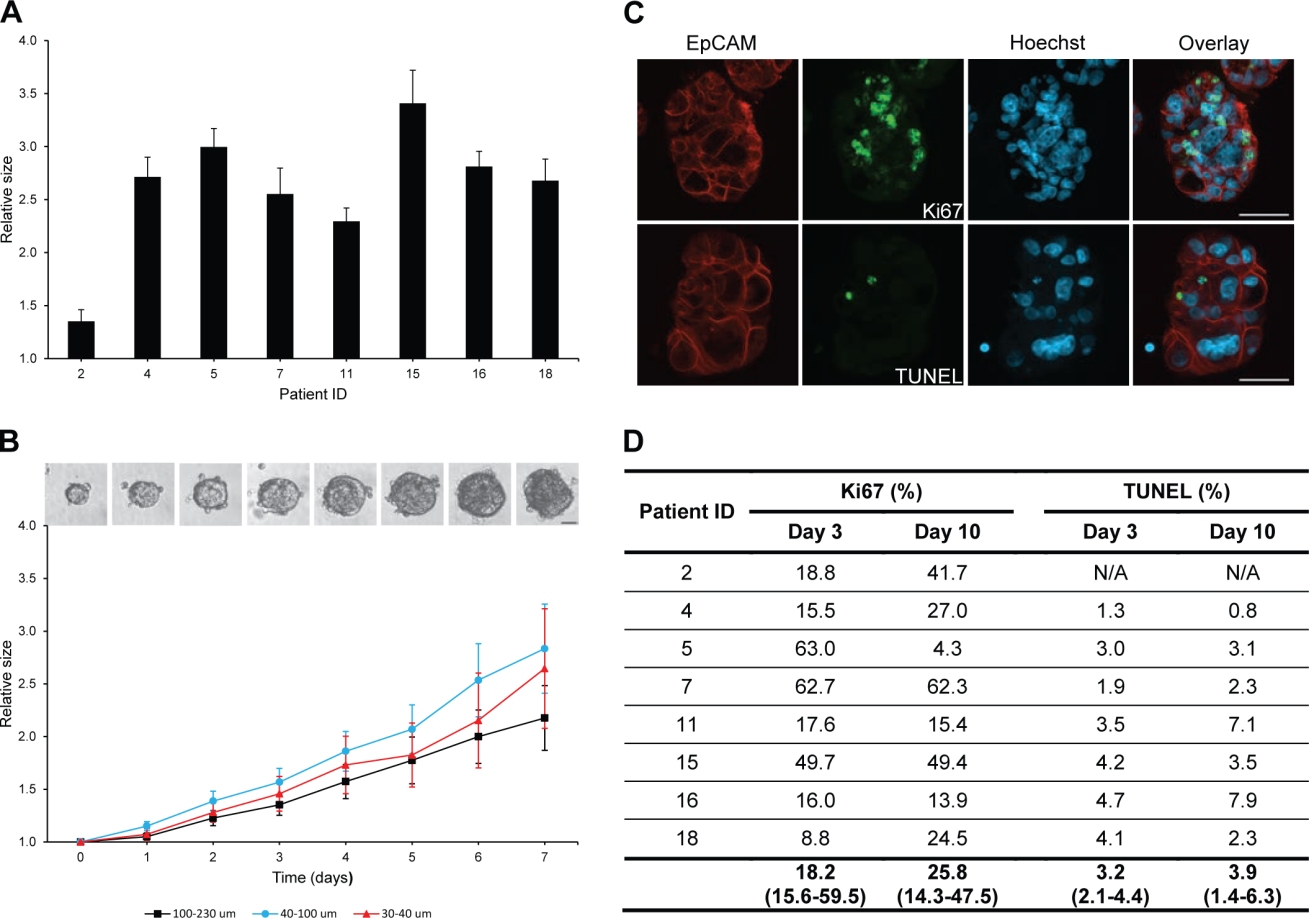
Spheroid growth and viability in short-term culture. (A) Significant growth of spheroid cultures from different tumours during 7 days of culture (p = 0.001). Growth of individual spheroids after 7 days was measured as the spheroid area on microscopic images with the area at baseline set to 1. Bars display mean values with standard error of the mean (SEM) for all three sizes of spheroids. (B) No significant difference in growth of different sizes of spheroids during 7 days of culture (p = 0.617). Growth of individual spheroids was measured as the spheroid area on microscopic images obtained every day with the area at baseline set to 1. Curves display mean values with SEM for one representative patient. Size bar = 50 μm. (C) Immunostaining of spheroids for epithelial cell marker EpCAM (red), active proliferation marker Ki67 (green) and apoptotic assay TUNEL (green) after 10 days of culture. Nuclei are stained with Hoechst (blue). Size bars = 50 μm. (D) No significant difference in percentage of EpCAM-positive spheroid cells stained for Ki67 (p = 1.000) and TUNEL (p = 0.454) at day 3 and day 10 was observed. Ki67: Median with interquartile range. TUNEL: Mean with 95% CI.

Growth of individual spheroids was monitored over 7 days. All spheroid cultures demonstrated a significant increase in relative size over a period of 7 days (p = 0.001) (Fig 2A). Spheroid growth for different cultures ranged from 1.4-fold to 3.4-fold increase in size and growth for all cultures averaged 2.6-fold increase in size. No significant difference in average growth was observed for the three different spheroid sizes (Fig 2B).

Spheroid sections were assessed for active proliferation by immunostaining for the proliferation marker Ki67 and apoptosis by TUNEL staining (Fig 2C). The percentage of EpCAM-positive cells expressing Ki67 at day 3 varied from 9% to 63% for different spheroid cultures (Fig 2D). Overall, no significant change in the percentage of Ki67-positive cells could be detected from day 3 to day 10 in culture. Very few apoptotic cells could be detected in the established spheroid cultures. On average, less than 5% of the EpCAM-positive cells stained with TUNEL (Fig 2D). The percentage of apoptotic cells did not change significantly from day 3 to day 10 in culture.

### Spheroid culture preserves colorectal adenocarcinoma histology

**Fig 3:**
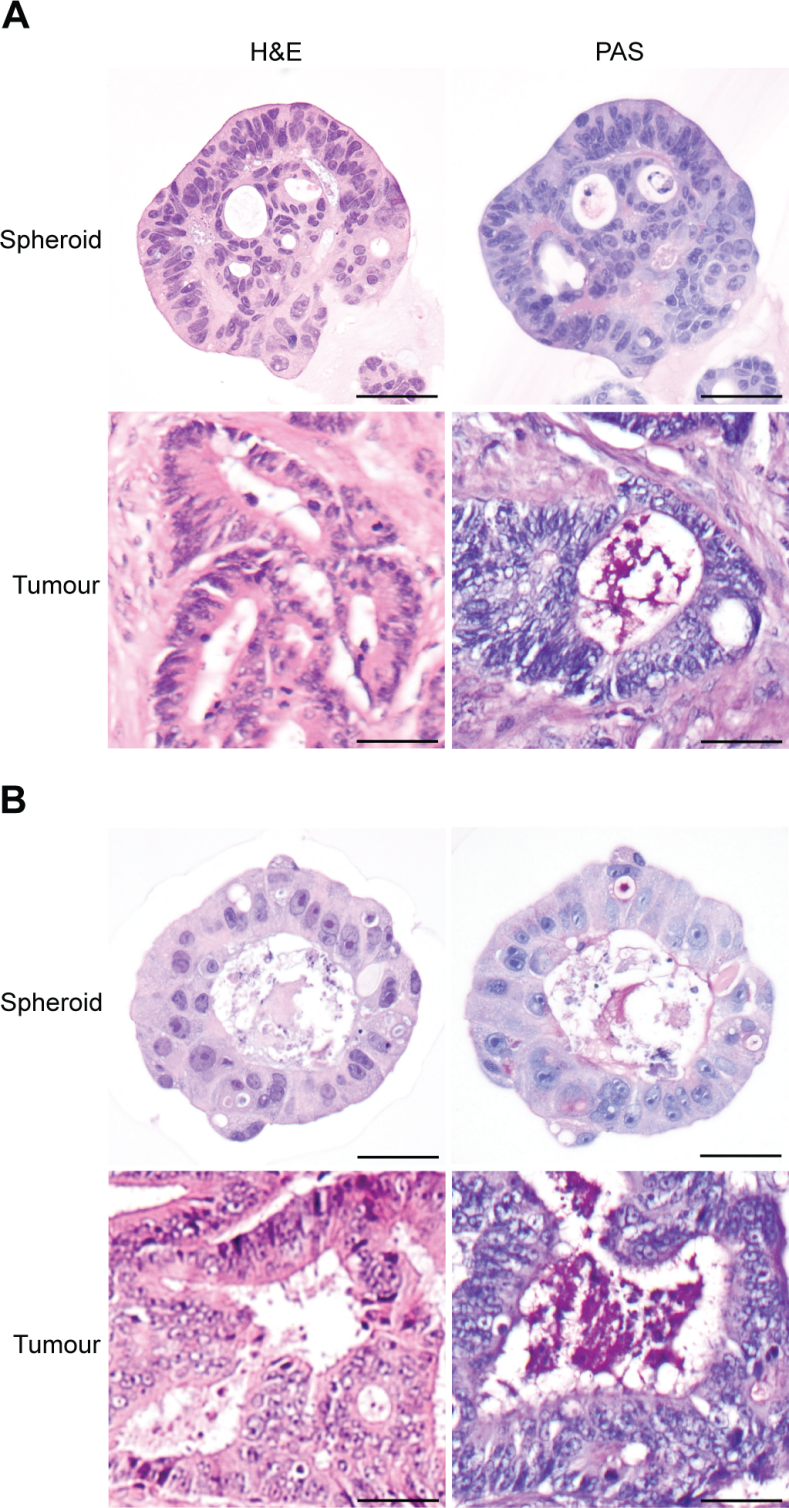
Spheroid cultures preserve cytology and histology of their original tumours. (A-C) H&E and PAS staining of spheroids and corresponding tumours from three different patients. Spheroids were stained after 10 days of culture. Size bars = 50 μm.

H&E staining confirmed that spheroids consisted of cells displaying classical neoplastic features, such as nuclear pleomorphism, increased nucleus/cytoplasm ratio, hyperchromasia and prominent nucleoli (Fig 3A and 3B). Spheroid cells were organised in glandular-like structures with polarized, aligned nuclei and luminal areas, and the degree of glandular organisation in spheroids generally reflected the original tumour. PAS staining demonstrated that pink mucin-producing cells were present to varying degree in spheroids from different tumours (Fig 3A and 3B). In some spheroids, mucin secretion into luminal areas could be observed (Fig 3B).

### Primary colorectal spheroids preserve protein expression patterns of the original tumour

**Fig 4:**
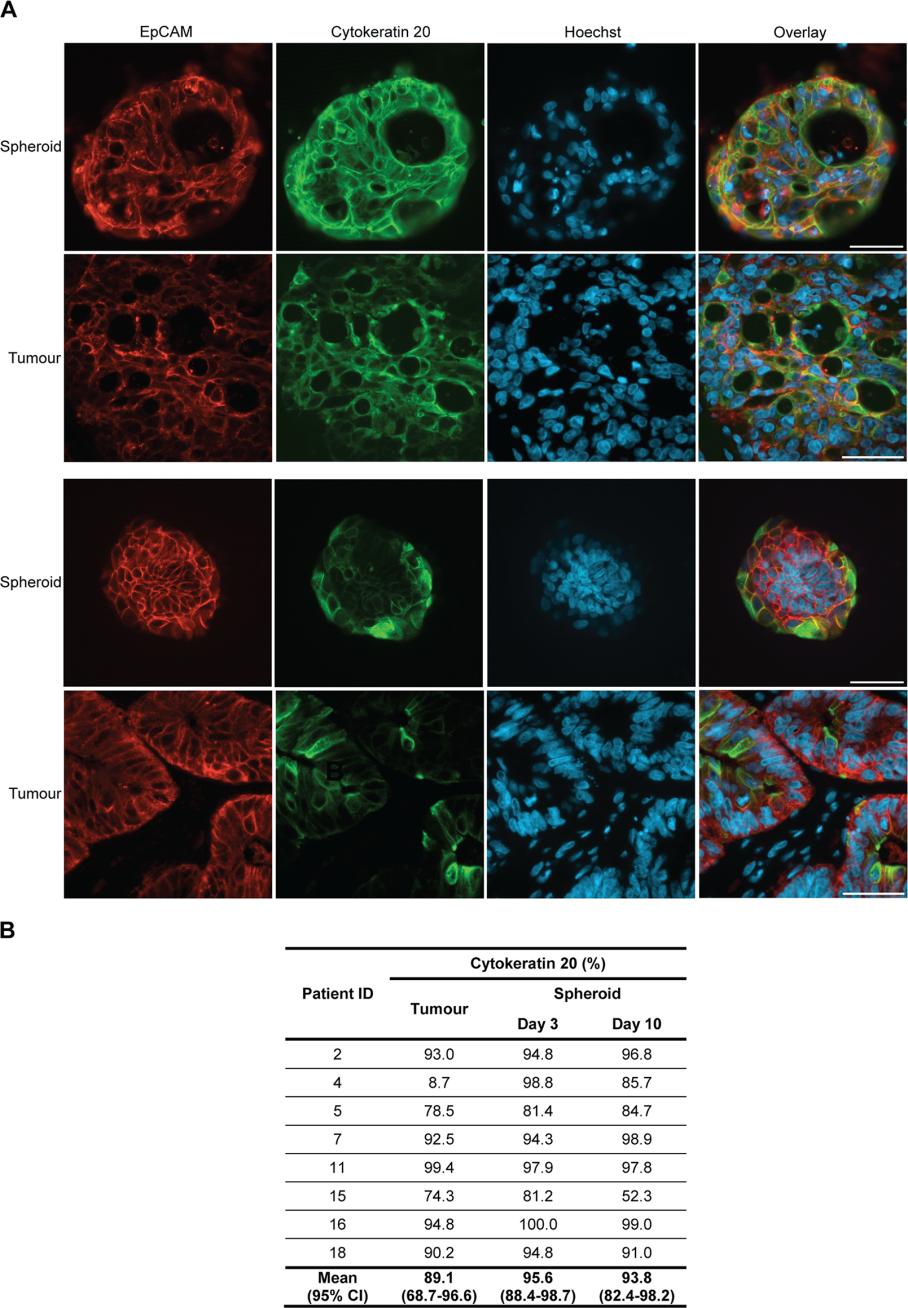
Spheroid cultures preserve cytokeratin 20 expression of their original tumours. (A) Immunostaining of spheroids and corresponding tumours from two different patients for epithelial cell marker EpCAM (red) and gastrointestinal epithelial marker cytokeratin 20 (green). Nuclei are stained with Hoechst (blue). Spheroids were stained after 10 days of culture. Size bars = 50 μm. (B) No significant difference in percentage of EpCAM-positive cells stained for cytokeratin 20 in tumours and spheroid cultures at day 3 and day 10 (p = 0.149) was observed.

Cytokeratin 20 is a cytoskeletal protein that is expressed by intestinal epithelial cells, especially those of the colon [34]. Immunostaining revealed that cytokeratin 20 was expressed to a varying degree by spheroid cultures established from different tumours (Fig 4A). For some cultures, variation in cytokeratin 20 expression for individual spheroids obtained from the same tumour could be observed. This variation reflected expression in different regions of the original tumour (S1 Fig). Overall, the average percentage of positive cells in the original tumour and the derived spheroids was comparable (Fig 4B). The percentage of EpCAM-positive cells expressing cytokeratin 20 was not significantly different between sections from the original tumour, spheroids at day 3 and spheroids at day 10. A few of the established cultures deviated from the original tumour; spheroids from patient 4 had a much higher expression of cytokeratin 20, while spheroids from patient 15 showed somewhat lower cytokeratin 20 expression at day 10.

**Fig 5:**
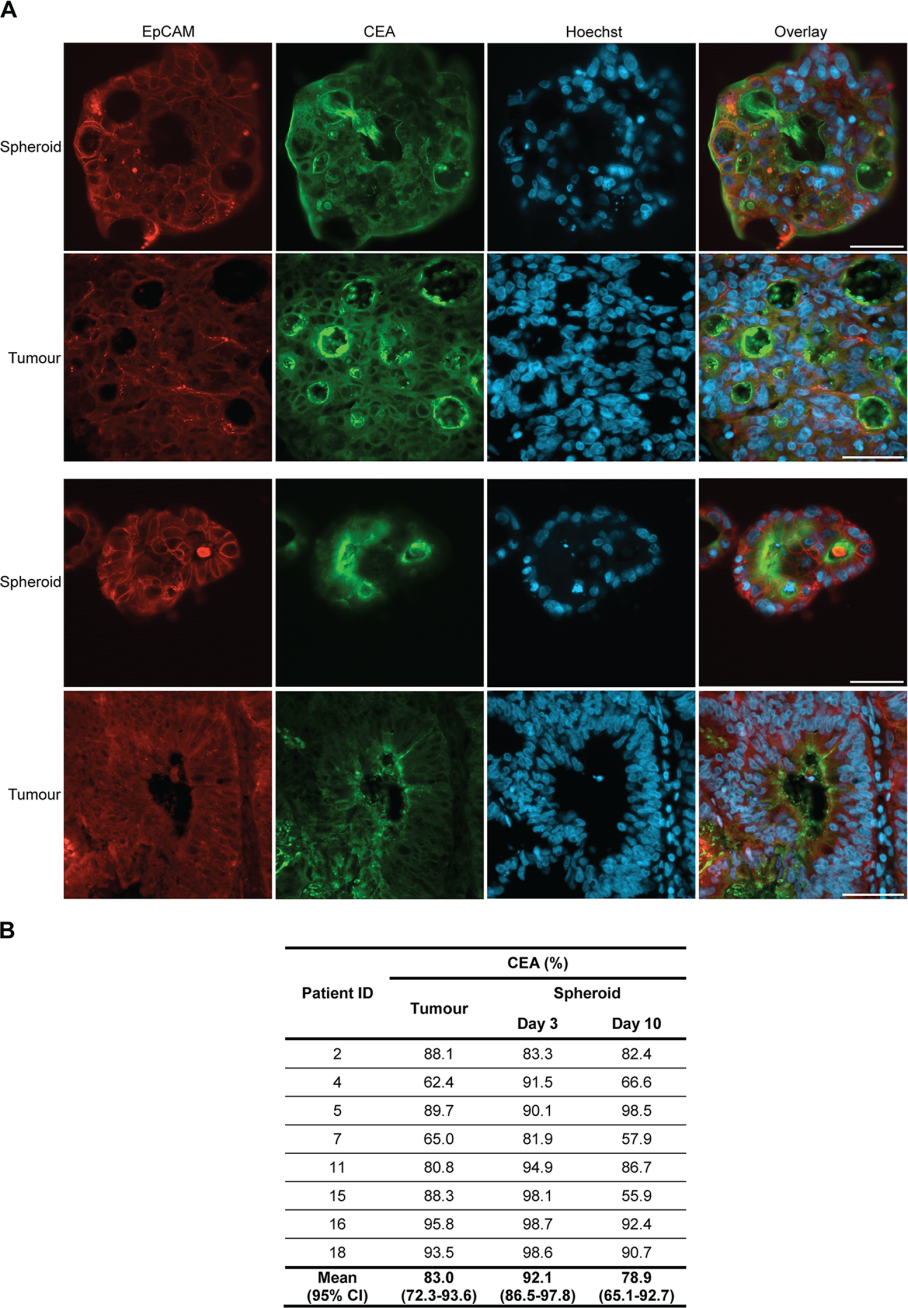
Spheroid cultures preserve CEA expression of their original tumours. (A) Immunostaining of spheroids and corresponding tumours from two different patients for epithelial cell marker EpCAM (red) and adenocarcinoma marker CEA (green). Nuclei are stained with Hoechst (blue). Spheroids were stained after 10 days of culture. Size bars = 50 μm. (B) Significant difference in percentage of EpCAM-positive cells stained for CEA in tumours and spheroid cultures at day 3 and day 10 (p = 0.040), but Bonferroni corrected post hoc analysis did not reach statistical significance (tumour vs. day 3 p = 0.139, tumour vs. day 10 p = 1.000 and day 3 vs. day 10 p = 0.158).

CEA is a glycoprotein that is often overexpressed in epithelial cancers, including colorectal carcinoma [35]. The spheroid cultures exhibited variations in their CEA expression patterns and levels (Fig 5A). Some cultures showed variation in CEA expression for individual spheroids which resembled expression patterns in different regions of the original tumours (S2 Fig). For most patients, expression in tumour tissue and spheroid cultures was comparable (Fig 5B). However, for several spheroid cultures expression at day 3 was higher than both the expression in the original tumour and at day 10. Overall, a significant difference in the percentage of EpCAM-positive cells expressing CEA in tumour sections, spheroids at day 3 and spheroids at day 10 was detected (p = 0.040), but Bonferroni corrected post hoc analysis did not reveal any significant differences when performing pairwise comparisons. The obtained results indicated that the percentage of cells expressing CEA was higher at day 3 compared to the tumour and day 10, but the differences did not reach significance.

### Individual spheroid cultures exhibit variation in chemosensitivity

**Fig 6:**
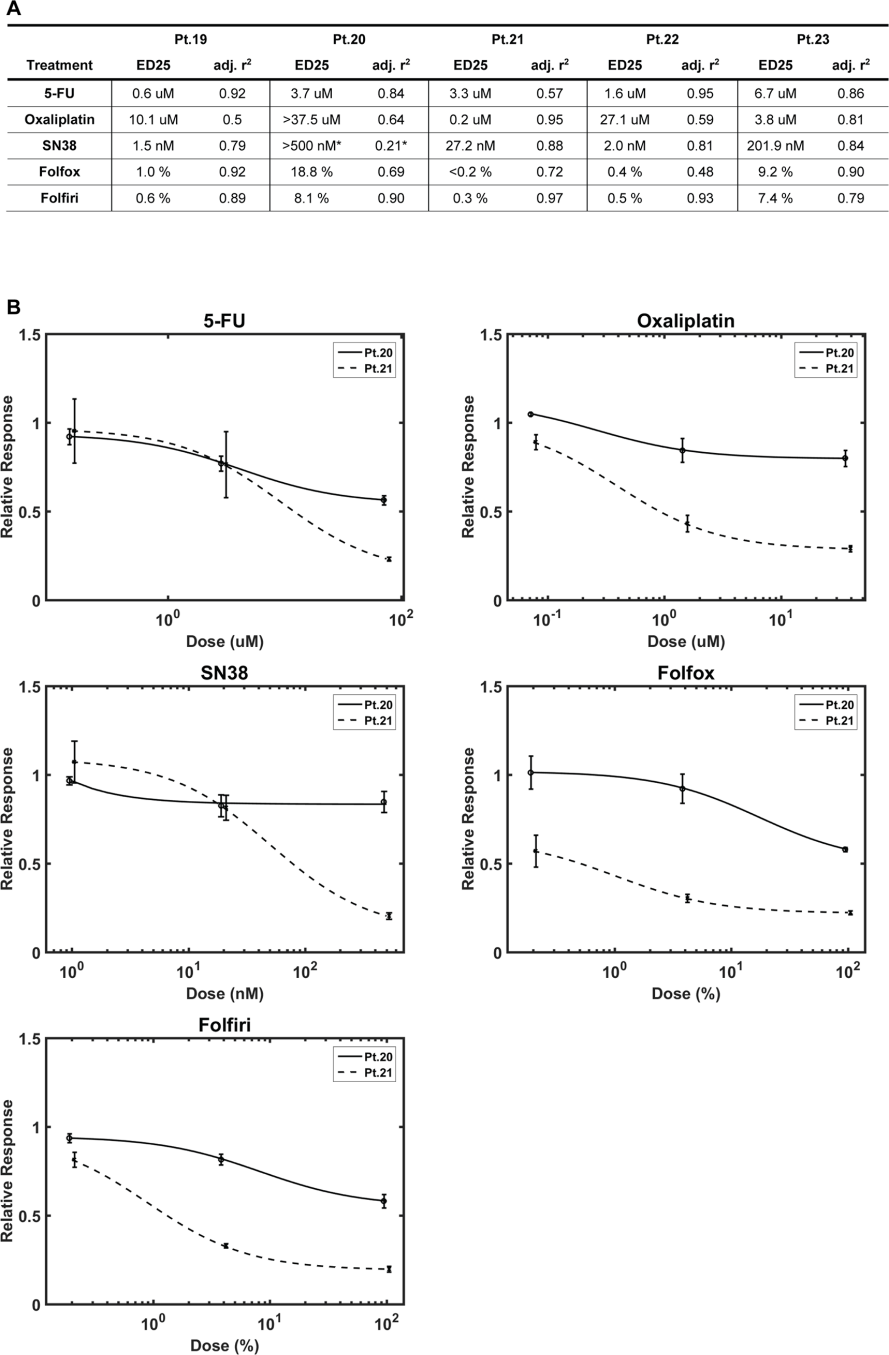
Chemosensitivity screening is possible using spheroid cultures. (A) Screening results for spheroid cultures exposed to common chemotherapy drugs and combinations from five patients. ED25: the dose at which a given drug resulted in 25% growth reduction compared to untreated spheroids. * poor curve fit, however, max growth reduction was 17% so ED25 was assigned >500nM. (B) Curve fit graphs for Pt.20 and Pt.21 demonstrating different chemosensitivity profiles.

The chemosensitivity of spheroids from five tumour samples were tested using IndiTreat™ arrays. Dose dependent inhibition of spheroid growth was observed for all the investigated drugs (5-FU, oxaliplatin and SN38) and combination treatments (FOLFOX and FOLFIRI). The resulting dose response curves and calculated ED25 values showed clear differences in sensitivity towards the different chemotherapeutic drugs. ED25 values for all of the screened cultures are shown in the table in Fig 6A. Dose response curves for two representative cultures are shown in Fig 6B. One of the examined cultures displayed differential sensitivity to the chemotherapeutic drugs (patient 22). Two of the spheroid cultures demonstrated high sensitivity to all the tested drugs (patient 19 and 21), whereas the two remaining cultures were generally more resistant (patient 20 and 23).

Several of the spheroid cultures displayed either general sensitivity or resistance to all the tested drugs. This could be caused by multidrug resistance mechanisms, such as drug efflux by ATP-dependent pumps [36]. The ATP-binding cassette transporters ABCG2 and ABCB1 have previously been associated with chemotherapy resistance, early disease recurrence and shorter survival in colorectal cancer [37–39]. Immunostaining of the original tumours demonstrated that the cancer cells expressed ABCG2 in all five tumours, but to varying extent (Panel A in S3 Fig). Four out of five examined colorectal tumours expressed ABCB1, however only few positive cancer cells were detected (Panel B in S3 Fig). Neither ABCG2 nor ABCB1 expression seemed to reflect overall chemosensitivity of the derived spheroid cultures.

## Discussion

Development of functional assays for predicting chemosensitivity of individual tumours is needed to improve clinical response rates. 3D culture of cancer cells is considered to reflect the *in vivo* tumour conditions more closely than conventional 2D culture and therefore represents a promising system for chemosensitivity testing. In the current study, colorectal cancer cells were isolated as tumour fragments and cultured in 3D as spheroids. Characterisation of the spheroids showed that important properties of the original tumour were retained during short-term (i.e. up to 10 days) *in vitro* culture. Importantly, spheroid cultures displayed heterogeneous response profiles when exposed to chemotherapy.

Spheroid cultures were successfully established in 83% of the colorectal adenocarcinoma samples. Staining confirmed that the spheroids consisted of epithelial-derived neoplastic cells. Two previous studies have reported a high overall success rate in establishing spheroid suspension cultures (99% and 89%) from resected colorectal tissue [31,33], while a third study only reported a success rate approximating 50% [30]. Differences in definition of “a successful culture” might explain some of the variation in success rates. Furthermore, the media composition might affect the success rate. Like the two studies reporting high success rates, we established cultures in serum-free medium, whereas the study with low culture success used serum-containing medium. This indicates that serum-free medium is more efficient for forming colorectal spheroids. In the study by Ashley et al., addition of ROCK1 inhibitor to the culture medium increased the success rate dramatically both in terms of establishing (from 75% to 100%) and maintaining cultures short-term (from 46% to 89%) [33]. ROCK1 inhibitor has been shown to reduce dissociation-induced apoptosis, known as anoikis, in both embryonic stem cell cultures and primary intestinal cultures [40,41]. Even though we did not add any inhibitors to the culture media, our success rate was comparable to the achieved short-term culture success in the study using ROCK1 inhibitor.

As in previous spheroid studies, we observed that established cultures predominantly consisted of epithelial-derived cells [30,31,33,42]. This is important, because overgrowth by fibroblasts remains one of the major challenges when culturing primary colorectal cancer cells in conventional 2D culture [43,44]. Immunostaining for the fibroblast marker TE-7 showed that only few fibroblasts were found in the spheroid cultures and no increase in fibroblasts was observed during culture. Studies have shown that fibroblasts grow poorly under serum-free culture conditions [45–47] which could explain the low number of fibroblasts detected in our spheroids. As more than 95% of cells in the spheroids stained for EpCAM and the percentage of EpCAM positive cells did not decrease with time in culture, staining for other stromal cells was not performed. In line with our observations, previous studies only detected few fibroblasts, endothelial and immune cells in primary colorectal spheroids [31,33,42], but the cell composition of spheroids over time was not studied.

A significant increase in spheroid size could be observed over a 7 day period for all the studied cultures. Interestingly, spheroid growth rate did not depend on size of the isolated tumour fragments, at least not within the investigated range. These results indicate that all the investigated spheroid sizes can be used for assaying growth and growth inhibition as a measure of drug sensitivity. Variation in growth rate was evident for cultures from different tumours, indicating that some intertumour heterogeneity was preserved. During 10 days of culture, active proliferation persisted in spheroids and very limited apoptosis was detected. Taken together, these data show that the primary spheroids remain viable for at least 10 days in the tested culture system.

In line with previous studies [31,33,42,48,49], we have demonstrated that primary colorectal 3D cultures display a number of features found in the original tumours. Histological staining of established cultures confirmed that spheroids retained characteristic features of colorectal adenocarcinomas. Furthermore, the degree of glandular organisation was similar to the original tumours.

A previous study also identified crypt-like structures in their primary colorectal spheroids [31]. Like in the present study, epithelial organisation was preserved by isolating cancer cells as fragments of tumour tissue that maintained cell-to-cell contacts. Many tumours display inherent molecular heterogeneity, and therefore random dissociation of tumour tissue might generate a heterogeneous population of spheroids. In our study, cultures derived from tumours with varying expression of cytokeratin 20 or CEA, also demonstrated variation in expression for individual spheroids. However, average expression in spheroid cultures was comparable with the average expression in the original tumours. Spheroid expression seemed to reflect the original tumour better after 10 days of culture than after 3 days. This is somewhat surprising, since it is well-established that cancer cells maintained in culture for longer time tend to become more deviating from their original tumour [50–52]. However, in this context 10 days is a relatively short time and molecular evolution might not have occurred yet. On day 3 the cultured cells might still be stressed from the isolation procedure performed on day 0. Tissue hypoxia, dissociation of tissue and artificial culture conditions can induce cellular stress responses that generate changes in e.g. protein expression and cell signalling [53–55]. Therefore, it might be important to consider the time since isolation when conducting experiments with spheroids.

We have demonstrated that chemosensitivity testing is possible using the IndiTreat™ screening array combined with tumour spheroids derived from both primary colorectal tumours and metastases. The results from five screens show varying sensitivities towards the chemotherapeutic drugs most commonly used for treatment of colorectal cancer. To our knowledge, this is the first time all the drugs used for first-line treatment of colorectal cancer (both as mono and combination therapies) have been tested on patient-derived tumour spheroids. Our results are in line with previous studies that also have observed variation in the sensitivity of 3D cultures derived from different colorectal tumours [31,42,48]. This coincides well with the heterogeneity of response seen when patients are treated with chemotherapy, indicating that chemosensitivity testing on tumour-derived spheroids may be useful in treatment selection. Importantly, the short time from sample to result (10–14 days) presented in the current study means that treatment selection can be accomplished in a patient-relevant timeframe. Van de Wetering et al. [48] observed organoid cultures displaying general sensitivity or resistance to chemotherapy. In agreement with their results, two of the spheroid cultures examined in the present study exhibited generally higher sensitivity to all the tested drugs. Two drug resistance mechanisms relevant to 3D culture are lack of drug penetration [56] or active drug efflux by e.g. ATP-dependent pumps [36]. The spheroids used for chemosensitivity screening were all of similar size. Consequently, the observed differences in drug sensitivity are not likely to be caused by size-mediated variance in drug penetration. Likewise, ABCG2 and ABCB1 expression levels in the original tumours did not explain the multidrug resistance observed in the derived cultures. This supports that functional *in vitro* characterisation of cancer cell chemosensitivity provides additional information to biomarker profiling of the tumour tissue.

In conclusion, primary colorectal spheroids generated in the present study successfully maintained histology and protein expression patterns of their original tumours. Our screening results indicate that patient-to-patient differences in response to chemotherapy are present in the spheroid cultures. Short-term spheroid culture of patient-derived cancer cells therefore represents a promising *in vitro* model for use in individualized medicine. Further studies are needed to determine how spheroid cultures functionally relate to the original tumour, especially in terms of chemosensitivity. To facilitate this, we are currently performing an interventional study to validate the screening system. In addition, more detailed investigation of intratumour heterogeneity and how this translates to the established spheroid cultures would be of clinical interest.

## Supporting information

S1 Table Overview of included patients. All tumours were classified as adenocarcinomas, except for one mucinous adenocarcinoma denoted with a *. MSI: microsatelite instability, MSI was not determined for liver metastasis (pt: 21, 22 & 23), M: male, F: female.
(DOC)

S1 Fig Spheroid cultures and their original tumours display heterogeneity in cytokeratin 20 expression. (A) Immunostaining of spheroids and different tumour areas from the same patient for epithelial cell marker EpCAM (red) and gastrointestinal epithelial marker cytokeratin 20 (green). Nuclei are stained with Hoechst (blue). Spheroids were stained after 10 days of culture. Size bars = 50 μm.
(TIF)

S2 Fig Spheroid cultures and their original tumours display heterogeneity in CEA expression. (A) Immunostaining of spheroids and different tumour areas from the same patient for epithelial cell marker EpCAM (red) and adenocarcinoma marker CEA (green). Nuclei are stained with Hoechst (blue). Spheroids were stained after 10 days of culture. Size bars = 50 μm.
(TIF)

S3 Fig Heterogeneous expression of ABCG2 and ABCB1 in colorectal tumours. (A) Immunostaining for epithelial cell marker EpCAM (red) and ATP-binding cassette transporter ABCG2 (green). Nuclei are stained with Hoechst (blue). (B) Immunostaining for epithelial cell marker EpCAM (red) and ATP-binding cassette transporter ABCB1 (green). Nuclei are stained with Hoechst (blue). Size bars = 50 μm.
(TIF)
