# Supplementary material for: Short-term spheroid culture of primary colorectal cancer cells as an in vitro model for personalizing cancer medicine
Source: PLoS One. 2017 Sep 6;12(9):e0183074. doi: 10.1371/journal.pone.0183074 (PMC5587104; doi:10.1371/journal.pone.0183074)
Supplement: S1 Table — All tumours were classified as adenocarcinomas, except for one mucinous adenocarcinoma denoted with a *. MSI: microsatelite instability, MSI was not determined for liver metastasis (pt: 21, 22 & 23), M: male, F: female. (DOC) [file pone.0183074.s001.doc]

| **Patient ID** | **Stage** | **Tumor location** | **MSI** | **Gender** | **Age (years)** |
| --- | --- | --- | --- | --- | --- |
| 1 | II | Transverse colon | No | F | 68 |
| 2 | II | Descending colon | No | F | 68 |
| 3 | II | Descending colon | Yes | F | 57 |
| 4 | II | Cecum | No | M | 83 |
| 5 | II | Cecum | Yes | M | 67 |
| 6* | II | Cecum | Yes | M | 72 |
| 7 | II | Rectum/sigmoid colon | No | M | 61 |
| 8 | II | Rectum | No | M | 76 |
| 9 | III | Ascending colon | No | M | 66 |
| 10 | III | Ascending colon | No | F | 66 |
| 11 | III | Cecum | No | M | 86 |
| 12 | III | Cecum | Yes | F | 72 |
| 13 | III | Sigmoid colon | No | M | 73 |
| 14 | III | Sigmoid colon | No | M | 72 |
| 15 | IV | Ascending colon | No | M | 63 |
| 16 | IV | Sigmoid colon | No | M | 72 |
| 17 | IV | Rectum/sigmoid colon | No | M | 78 |
| 18 | IV | Rectum | No | M | 48 |
| 19 | I | Ascending colon | Yes | M | 71 |
| 20 | II | Rectum | Yes | F | 76 |
| 21 | IV | Liver | - | F | 72 |
| 22 | IV | Liver | - | F | 52 |
| 23 | IV | Liver | - | F | 77 |
