# Supplementary figures and images for: Short-term spheroid culture of primary colorectal cancer cells as an in vitro model for personalizing cancer medicine
Source: PLoS One. 2017 Sep 6;12(9):e0183074. doi: 10.1371/journal.pone.0183074 (PMC5587104; doi:10.1371/journal.pone.0183074)

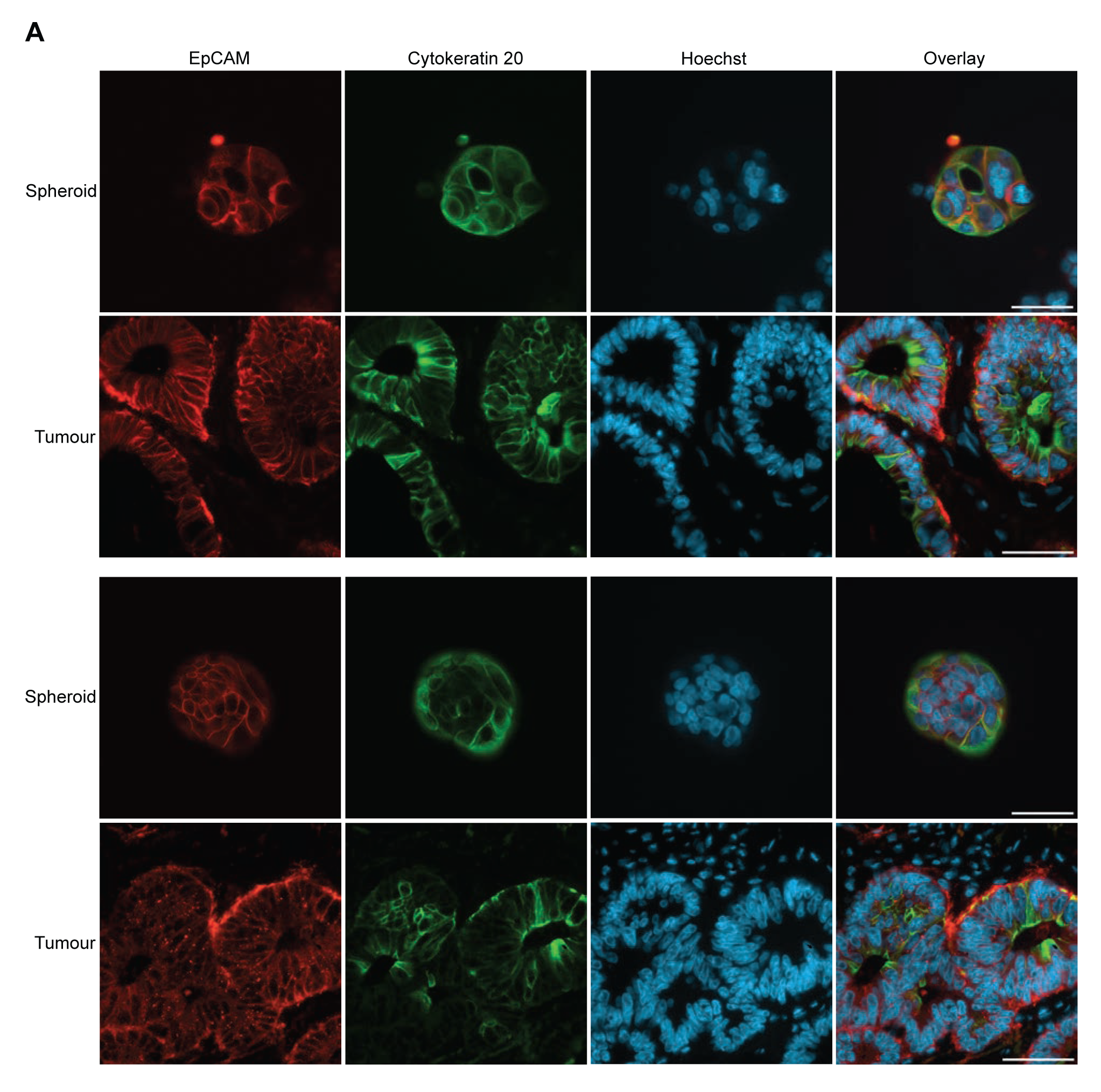

Supplement: S1 Fig — (A) Immunostaining of spheroids and different tumour areas from the same patient for epithelial cell marker EpCAM (red) and gastrointestinal epithelial marker cytokeratin 20 (green). Nuclei are stained with Hoechst (blue). Spheroids were stained after 10 days of culture. Size bars = 50 μm. (TIF) [file pone.0183074.s002.tif]

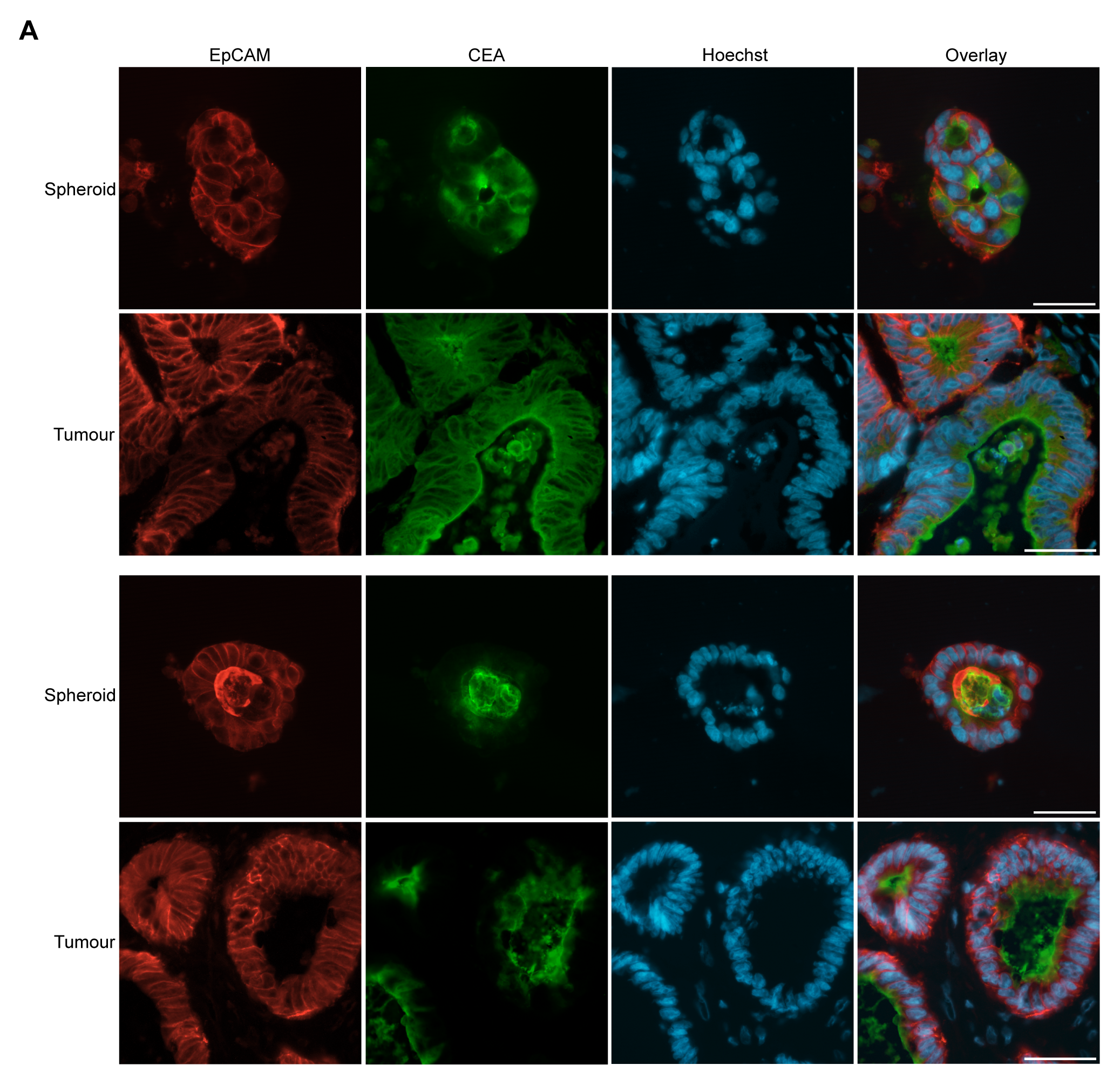

Supplement: S2 Fig — (A) Immunostaining of spheroids and different tumour areas from the same patient for epithelial cell marker EpCAM (red) and adenocarcinoma marker CEA (green). Nuclei are stained with Hoechst (blue). Spheroids were stained after 10 days of culture. Size bars = 50 μm. (TIF) [file pone.0183074.s003.tif]

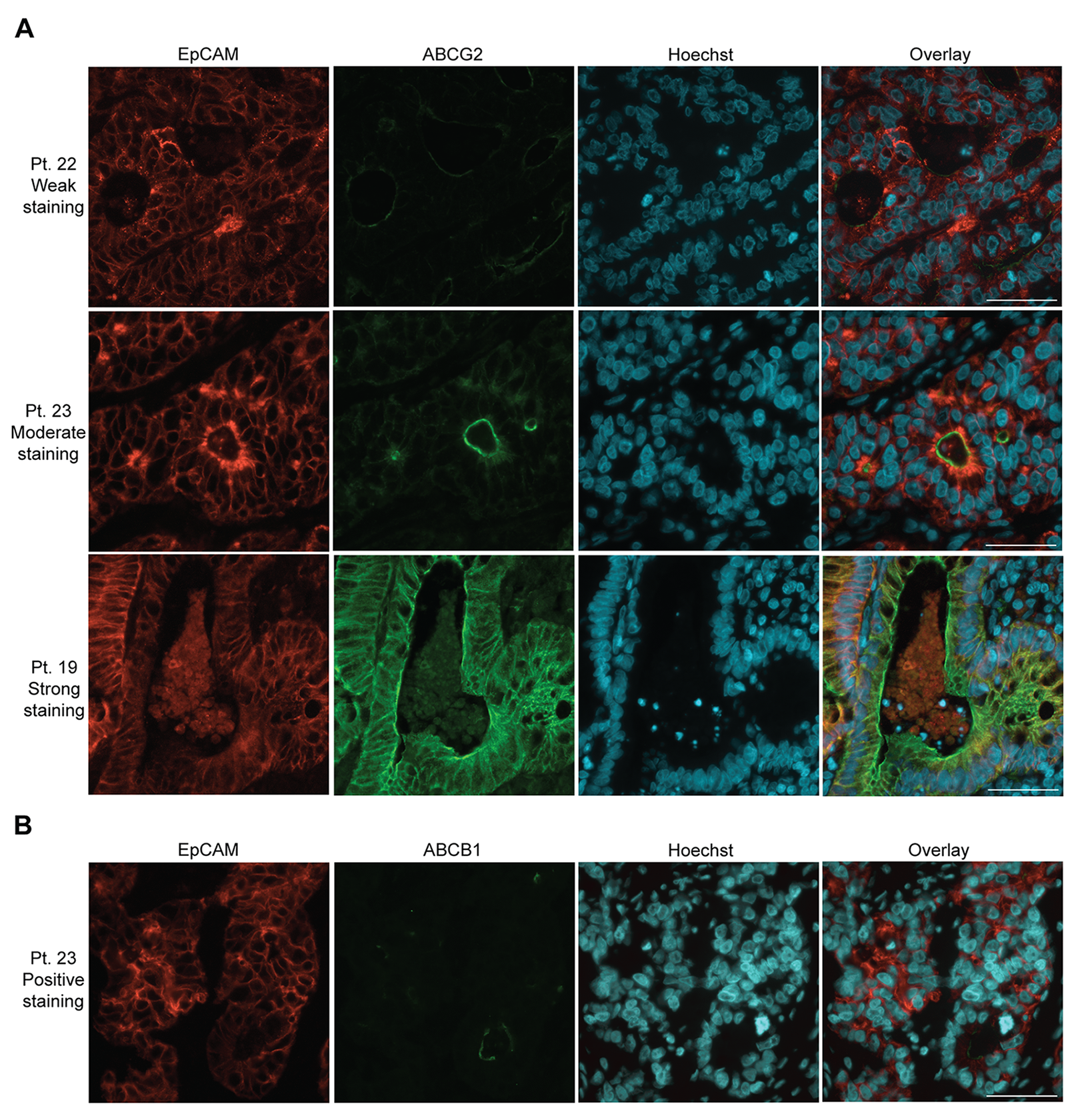

Supplement: S3 Fig — (A) Immunostaining for epithelial cell marker EpCAM (red) and ATP-binding cassette transporter ABCG2 (green). Nuclei are stained with Hoechst (blue). (B) Immunostaining for epithelial cell marker EpCAM (red) and ATP-binding cassette transporter ABCB1 (green). Nuclei are stained with Hoechst (blue). Size bars = 50 μm. (TIF) [file pone.0183074.s004.tif]
